# Promoter of Cassava *MeAHL31* Responds to Diverse Abiotic Stresses and Hormone Signals in Transgenic *Arabidopsis*

**DOI:** 10.3390/ijms25147714

**Published:** 2024-07-14

**Authors:** Xiao-Tong Wang, Xiang-Ning Tang, Ya-Wen Zhang, Yu-Qiang Guo, Yuan Yao, Rui-Mei Li, Ya-Jie Wang, Jiao Liu, Jian-Chun Guo

**Affiliations:** 1 National Key Laboratory for Tropical Crop Breeding, School of Life and Health Sciences, Hainan University, Haikou 570228, China; wangxiaotong@hainanu.edu.cn (X.-T.W.); 22220860000067@hainanu.edu.cn (X.-N.T.); 21210710000021@hainanu.edu.cn (Y.-W.Z.); 20071000110021@hainanu.edu.cn (Y.-Q.G.); 2 National Key Laboratory for Tropical Crop Breeding, Sanya Research Institute, Institute of Tropical Bioscience and Biotechnology, Chinese Academy of Tropical Agricultural Sciences, Haikou 571101, China; yaoyuan@itbb.org.cn (Y.Y.); liruimei@itbb.org.cn (R.-M.L.); wangyajie@itbb.org.cn (Y.-J.W.)

**Keywords:** cassava, AT-hook protein, promoter, GUS, transgenic *Arabidopsis*, abiotic stresses, hormone signals, *MeAHL31*

## Abstract

The AT-hook motif nuclear-localized (*AHL*) family is pivotal for the abiotic stress response in plants. However, the function of the cassava *AHL* genes has not been elucidated. Promoters, as important regulatory elements of gene expression, play a crucial role in stress resistance. In this study, the promoter of the cassava *MeAHL31* gene was cloned. The MeAHL31 protein was localized to the cytoplasm and the nucleus. qRT-PCR analysis revealed that the *MeAHL31* gene was expressed in almost all tissues tested, and the expression in tuber roots was 321.3 times higher than that in petioles. Promoter analysis showed that the *MeAHL31* promoter contains drought, methyl jasmonate (MeJA), abscisic acid (ABA), and gibberellin (GA) *cis*-acting elements. Expression analysis indicated that the *MeAHL31* gene is dramatically affected by treatments with salt, drought, MeJA, ABA, and GA3. Histochemical staining in the *proMeAHL31-GUS* transgenic *Arabidopsis* corroborated that the GUS staining was found in most tissues and organs, excluding seeds. Beta-glucuronidase (GUS) activity assays showed that the activities in the *proMeAHL31-GUS* transgenic *Arabidopsis* were enhanced by different concentrations of NaCl, mannitol (for simulating drought), and MeJA treatments. The integrated findings suggest that the *MeAHL31* promoter responds to the abiotic stresses of salt and drought, and its activity is regulated by the MeJA hormone signal.

## 1. Introduction

Cassava is the sixth largest tropical food crop in the world and serves as a vital industrial raw material [1]. Therefore, obtaining high-yield cassava becomes an important goal for cassava breeding. However, cassava yields are frequently compromised by adverse environmental conditions, including persistent drought and salt stress [2]. Currently, research on the abiotic stress response mechanisms of cassava is extremely inadequate. It is promising to identify genes responsive to environmental factors and develop cassava cultivars with enhanced tolerance to abiotic stresses, especially in light of the ongoing impacts of global climate changes.

The AT-hook motif nuclear-localized (*AHL*) gene family is widespread in bacteria, fungi, plants, and animals [3]. There are two conservative domain structures in this gene family: the AT-hook motif and the plant and prokaryote (PPC or domain of unknown function 296, DUF296) domain [4]. AT-hook motifs are divided into two types due to their consistent variation. The type I motif consists of a Gly-Ser-Lys-Asn-Lys (GSKNK) consistent sequence, while the type II motif consists of the core sequence Arg-Gly-Arg (RGR) and Arg-Lys-Tyr (RKY) at the C-terminal [5]. The PPC domain contains the conserved sequence Gly-Arg-Phe-Glu-Ile-Leu, which is about 120 amino acids in length and has the same secondary or tertiary structure from prokaryotes to higher plants. Based on sequence variation, PPC domains are divided into two categories: type A domain contains a conserved core sequence, Leu-Arg-Ser-His (LRSH), and type B domain contains the upstream region, Phe-Thr-Pro-His (FTPH) [6]. AHLs are classified into three subtypes and two clades based on the abundance and composition of the two structural units described above, which are type I, containing a type AT-hook motif and a type A PPC domain, belonging to Clade A. Type II contains two AT-hook motifs (type I and type II) and a type B PPC domain, which also belongs to the Clade B. Type III contains a type II AT-hook motif and a type B PPC domain, which belongs to Clade B. At present, studies on *AHLs* in plants mainly focus on the model plant *Arabidopsis thaliana*, with additional studies conducted on *Oryza sativa*, *Zea mays*, *Brassica napus*, *Populus trichocarpa*, *Gossypium hirsutum*, *Solanum lycopersicum*, *Glycine max* and *Poncirus trifoliata* [7,8,9,10,11,12,13,14,15,16,17]. *AHLs* are implicated in a multitude of biological processes, primarily in plant growth and development, organogenesis, hormonal signal transduction, and stress regulation [12,17,18]. For instance, *AHLs* can inhibit petiole growth by antagonizing *PIF* [18]. *AHLs* can also be involved in regulating the length of the proliferation zone and the number of dividing cells in the interroot meristem, and affect the development of both primary and lateral roots [12]. Besides, *AHLs* are crucial in enhancing plant resilience to abiotic stresses such as drought, cold, and high salinity [9,17,19,20,21,22]. However, research on the *AHL* genes in cassava remains limited.

Promoters play a crucial role in plants’ response to abiotic stress by harboring *cis*-acting elements that initiate the expression of stress-responsive genes. These elements, such as MYB binding sites (MBS), which play a role in the drought-inducibility of genes and ABA-responsive elements (ABRE), interact with specific transcription factors, thereby modulating the transcriptional activity of genes involved in stress tolerances [23,24,25,26,27,28]. *AHLs* play an important role in abiotic stresses, but little is known about how the expression of these genes is regulated by promoters. Meanwhile, stress-responsive promoters hold significant potential for application in plant genetic engineering, particularly for enhancing stress tolerance [29]. However, the functions of most *AHL* promoters remain largely unknown. It is beneficial to explore the roles of other functionally uncharacterized *AHL* promoters.

Hormonal signal transduction is essential for regulating plant responses to abiotic stresses [30]. The intricate network of hormonal signals allows plants to adapt to environmental challenges such as drought and salinity [31]. The levels of ABA and MeJA in apples are significantly and positively correlated with their drought resistance, suggesting that endogenous ABA and MeJA may influence this trait [32]. When exogenous hormones were applied under drought stress, they increased the endogenous hormone content, enhanced the activity of carbon metabolism enzymes, improved specific photosynthetic fluorescence characteristics of leaves, and regulated the source-sink balance, which collectively alleviated the yield reduction caused by drought stress in sweet potato [33]. However, the specific mechanisms linking hormone signal transduction to stress resistance in *AHL* genes in cassava remain unexplored.

*MeAHL31* (GenBank: QBA99483.1), was previously identified through a yeast one-hybrid screen by our research group. In this study, the unique promoter for the *MeAHL31* gene was extracted and defined from cassava. The study then explored the histochemical expression patterns and transcriptional response of the *MeAHL31* promoter when subjected to multiple abiotic stresses in transgenic *Arabidopsis*. These findings indicate that GUS activities driven by the *MeAHL31* promoter are detectable in most tissues and organs, and were remarkably enhanced by salt, drought, and MeJA stresses. These findings contribute to clarifying the transcriptional regulation mechanism of the *MeAHL31* gene and offer a valuable stress-responsive promoter resource for plant genetic engineering.

## 2. Results

### 2.1. Isolation and Characterization of MeAHL31

**Figure 1:**
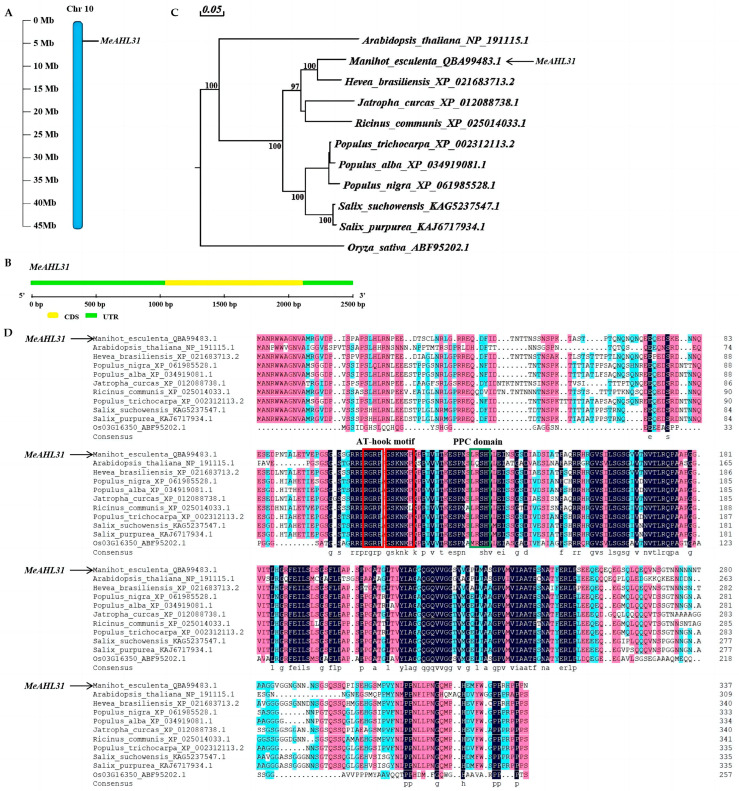
Chromosomic localization, gene structure of *MeAHL31* gene, conserved domain, and phylogenetic tree. (**A**) Chromosomic localization. (**B**) Exon–intron structure of the *MeAHL31* gene. (**C**) Phylogenetic tree of MeAHL31 and AHL proteins in other species. (**D**) Alignment of AHLs protein sequences in different species.

The CDS sequence of *MeAHL31* is 1014 bp in length, encoding 337 amino acids [34], and is located on chromosome 10 (Figure 1A). The exon–intron analysis indicates that *MeAHL31* has no introns and consists of only one exon (Figure 1B). The evolutionary relationship between *MeAHL31* and *AHLs* in other species indicates that its closest species is *Hevea brasiliensis*. It is also closely related to *Jatropha curcas* and *Ricinus communis*, but more distantly related to the monocotyledonous plant of the *Oryza sativa* Japonica group (Figure 1C). Sequence alignment of the MeAHL31 protein revealed the presence of AT-hook motif and PPC domains in the structure (Figure 1D). Further analysis demonstrates that the MeAHL31 protein is classified as an AHL type I (Figure 1D).

### 2.2. Subcellular Localization of MeAHL31 Protein

**Figure 2:**
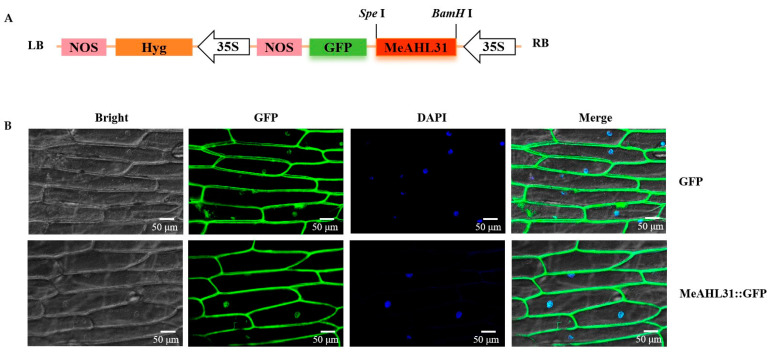
Subcellular localization of MeAHL31 protein in onion epidermal cells. (**A**) Diagrammatic illustration of the assembly for the pCAMBIA1300-35S-MeAHL31::GFP expression vector; LB refers to the left terminus and RB denotes the right terminus of the pCAMBIA1300-35S-GFP vector framework. (**B**) The observed fluorescence in the cells that underwent transfection. The images, in sequence, from left to right, display the bright field view, the GFP fluorescence, the DAPI-stained nuclear region, and the overlay of these fields. Bar = 50 µm.

To ascertain the subcellular localization of the MeAHL31 protein, we engineered a fusion expression vector, pCAMBIA1300-35S-MeAHL31::GFP, for transient expression in onion epidermal cells. A control vector, pCAMBIA1300-GFP, devoid of the MeAHL31 gene, was used for comparison. The findings revealed that the GFP fluorescence from the control was observable in both the cytoplasm and the nucleus. Similarly, the MeAHL31::GFP fluorescence also exhibited a similar distribution, indicating that the MeAHL31 protein is localized in both the cytoplasm and the nucleus (Figure 2).

### 2.3. Expression Patterns of MeAHL31 in Cassava Tissues

**Figure 3:**
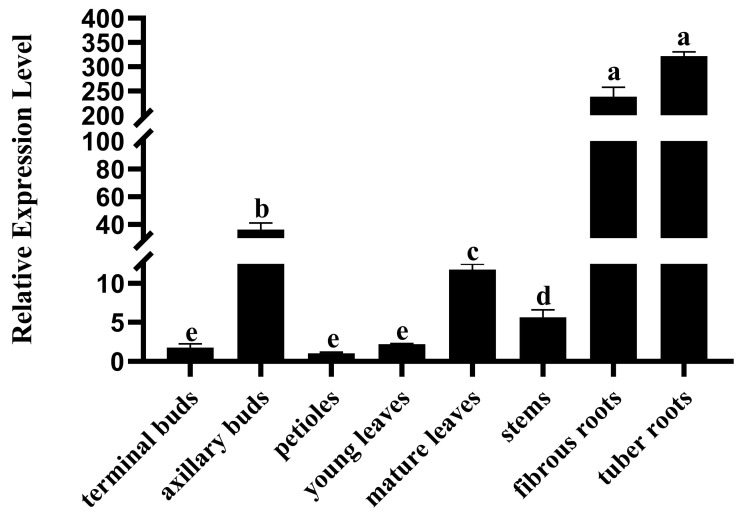
Expression analysis of MeAHL31 gene in cassava tissues. MeTublin and MeActin were used as internal controls. The expression levels of the petioles were set to a value of 1. Data represent means of three biological repeats ± standard deviations. Different lowercase letters indicated significant differences (*p* < 0.05).

The expression of the MeAHL31 gene in cassava tissues was analyzed by qRT-PCR. The results showed that the MeAHL31 gene was highly expressed in tuber roots and fibrous roots, and the expression levels were significantly higher than in other tissues (Figure 3).

### 2.4. Isolation and Cis-Acting Element Distribution of MeAHL31 Promoter

A 1.323 kb promoter sequence, located upstream of the ATG start codon of the *MeAHL31* gene, has been successfully cloned and named as *proMeAHL31* (Figure S1). The *cis*-acting elements of the *MeAHL31* promoter were identified using the PlantCARE database. It was found that *proMeAHL31* contains basic promoter elements, such as TATA, CATT boxes, and some light-responsive elements (Table S1). Numerous elements related to abiotic stresses and hormone signaling were discovered, including one drought stress response element (MBS), one ABA response element (ABRE), two MeJA response elements (CGTCA and TGACG motifs), and one GA response element (GARE) (Table 1). The results suggest that the *MeAHL31* promoter might be involved in diverse abiotic stresses and hormone-signaling responses.

### 2.5. Expression Patterns of MeAHL31 Gene under Abiotic Stresses and Hormone Treatments

**Figure 4:**
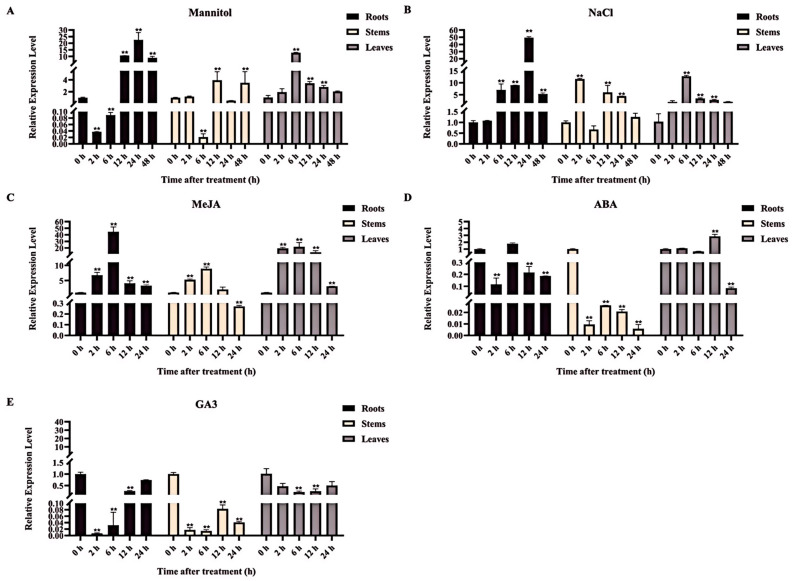
Transcriptional responses of the MeAHL31 gene to various stress conditions, including drought, salinity, methyl jasmonate (MeJA), abscisic acid (ABA), and gibberellic acid (GA), in the context of cassava seedling growth. Cassava seedlings were subjected to a series of stress treatments with the following concentrations: 100 mM mannitol for osmotic stress (**A**), 300 mM NaCl for salt stress (**B**), 100 µM MeJA to induce jasmonic acid response (**C**), 100 µM ABA to mimic the effects of abscisic acid (**D**), and 100 µM GA3 to represent gibberellin treatment (**E**). The *MeTublin* gene served as an endogenous reference for normalization. The relative expression values of the control samples were standardized to a reference value of 1. The data are presented as the average of three independent biological replicates, with error bars indicating the standard deviation from the mean. Each replicate consisted of six in vitro cultured cassava seedlings. An asterisk (**) denotes statistically significant differences from the control group at a *p*-value of less than 0.01 (*t*-test).

To confirm the roles of *MeAHL31* in response to abiotic stresses and hormone signals, the expression patterns of *MeAHL31* in cassava seedlings treated with mannitol, NaCl, MeJA, ABA, and GA3 were analyzed by qRT-PCR. Following a 100 mM mannitol treatment, the expression of *MeAHL31* reached the most dramatic induction (22.5 fold) at 24 h in roots and the most significant induction (12.7 fold) in leaves at 6 h (Figure 4A). However, *MeAHL31* expression presented no obvious change in stems (Figure 4A). After a 300 mM NaCl treatment, the *MeAHL31* expression was significantly induced at 24 h in roots (49.3 fold), 2 h in stems (11.5 fold), and 6 h in leaves (12.7 fold), respectively (Figure 4B). Upon treatment with 100 µM MeJA treatment, the expression of *MeAHL31* in roots, stems, and leaves was markedly up-regulated at 6 h by 44.4 fold, 8.9 fold, and 21.7 fold (Figure 4C). After a 100 µM ABA treatment, *MeAHL31* expression increased by 2.8 fold at 12 h in leaves (Figure 4D), while gene expression decreased in stems (Figure 4D). Exposure to a 100 µM GA3 treatment resulted in a downward trend in *MeAHL31* expression levels in roots, stems, and leaves (Figure 4E). The results indicated that *MeAHL31* may be responsive to stimuli from salt, drought, MeJA, ABA, and GA3.

### 2.6. Histochemical Localization of MeAHL31 Promoter

**Figure 5:**
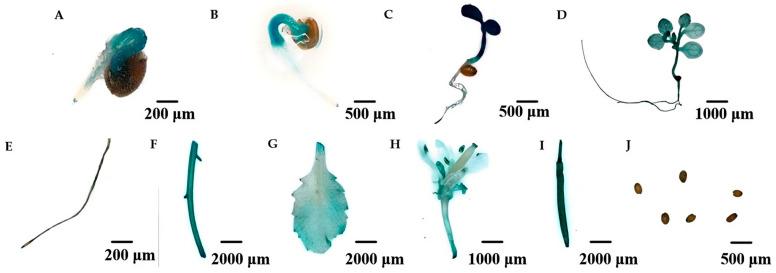
Tissue expression patterns of *MeAHL31* promoter in transgenic *Arabidopsis*. (**A**) Seed germination after 1 day. (**B**) Seed germination after 3 days. (**C**) Seed germination after 7 days. (**D**) Seedlings. (**E**) Roots. (**F**) Internodes. (**G**) Rosette leaves. (**H**) Flowers. (**I**) Siliques. (**J**) seeds.

To evaluate the function of the *MeAHL31* promoter, the pCAMBIA1304-*proMeAHL31*-GUS::GFP vector was constructed and transformed into *Arabidopsis* plants (Figure S2). GUS staining was observed in the transgenic seedlings, encompassing the roots, internodes, rosette leaves, flowers, and siliques (Figure 5D–I). Notably, GUS staining was absent in the seeds (Figure 5J), with staining only becoming evident after germination (Figure 5A–C). These observations indicated that the *MeAHL31* promoter plays a key role throughout the plant’s developmental stages.

### 2.7. Activities of MeAHL31 Promoter in Response to Salt and Drought Stresses

**Figure 6:**
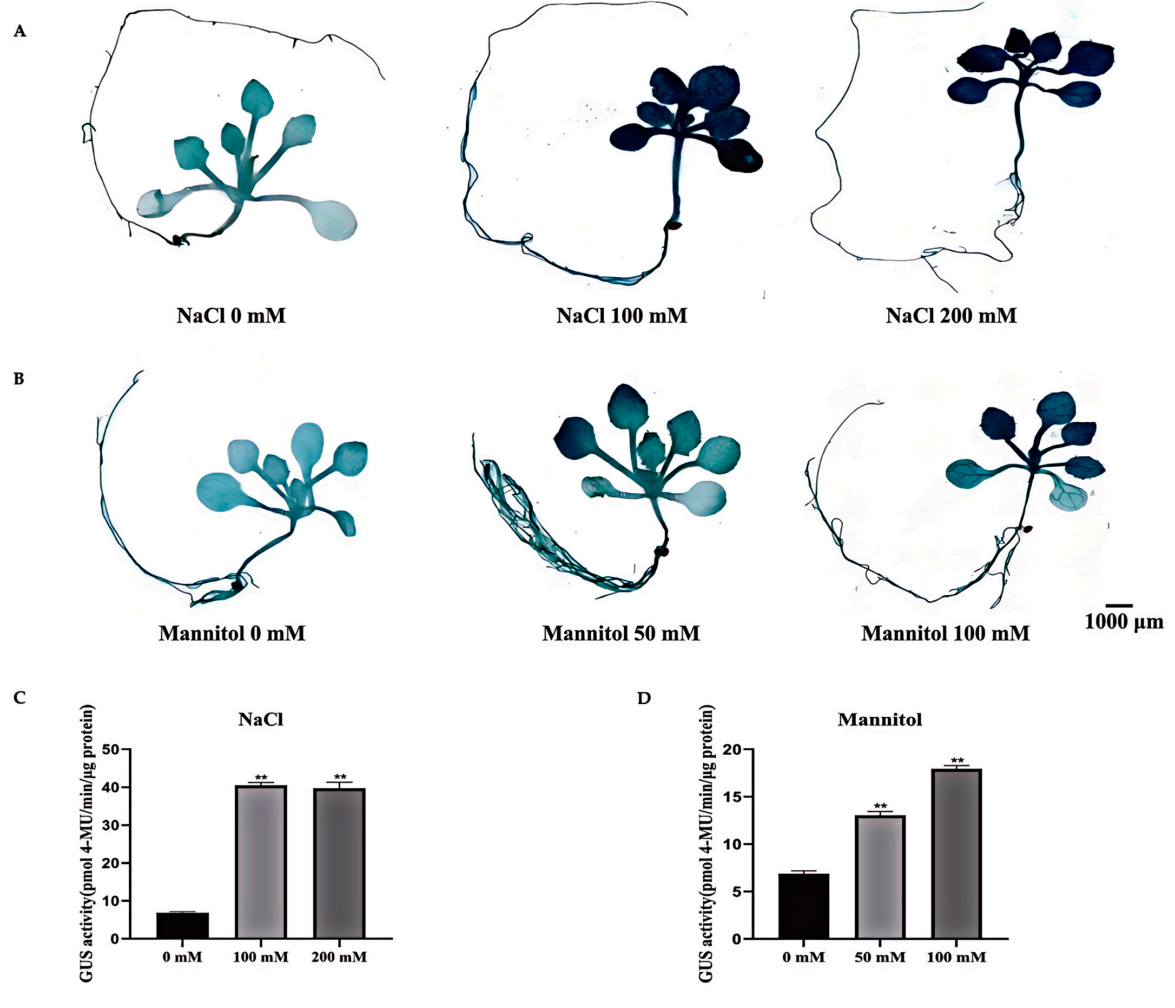
GUS activities of the *MeAHL31* promoter under salt and drought treatments in transgenic *Arabidopsis*. (**A**,**B**) GUS staining for the *proMeAHL31*-GUS transgenic seedlings treated with 100–200 mM NaCl or 50–100 mM mannitol. (**C**,**D**) Activity analyses of GUS protein in transgenic seedlings under 100–200 mM NaCl or 50–100 mM mannitol treatments. Data represent means of three biological repeats ± standard deviations. Each replicate was composed of six *Arabidopsis* seedlings. ** indicated significant differences in comparison with the control treatment, *p* < 0.01 (*t*-test).

To elucidate the roles of the *MeAHL31* promoter in response to salt and drought stresses, transgenic *Arabidopsis* seedlings were subjected to 100–200 mM NaCl or 50–100 mM mannitol, and the resulting GUS activities were compared. After NaCl treatment, GUS staining was sharply intensified (Figure 6A). Notably, there was no significant difference in GUS activity between the 100 mM and 200 mM NaCl (Figure 6B). Similarly, mannitol treatment led to a pronounced induction of GUS staining (Figure 6C). The GUS activity under 100 mM mannitol was stronger than that under 50 mM mannitol (Figure 6D). These results confirmed that the activity of the *MeAHL31* promoter could be elevated by both salt and drought stresses. 

### 2.8. Activities of MeAHL31 Promoter in Response to MeJA, ABA, and GA3 Signals

**Figure 7:**
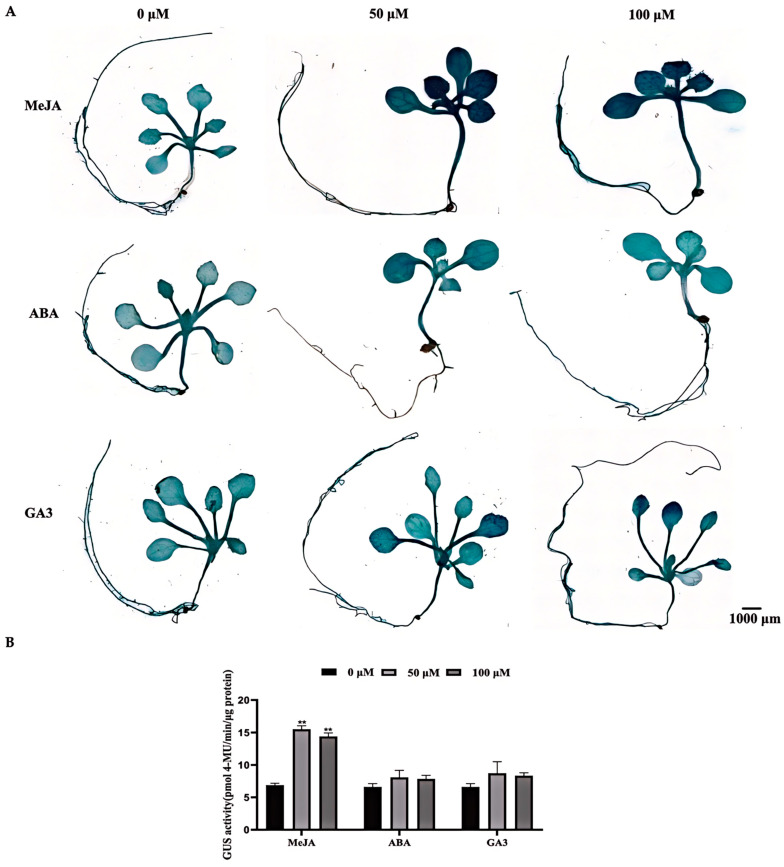
The β-glucuronidase (GUS) activity, as a measure of the MeAHL31 promoter’s activity, was evaluated in transgenic Arabidopsis when exposed to hormonal treatments of methyl jasmonate (MeJA), abscisic acid (ABA), and gibberellin (GA). (**A**) GUS staining for proMeAHL31-GUS transgenic seedlings treated with different hormone signals (50–100 µM MeJA or 50–100 µM ABA or 50–100 µM GA3). (**B**) GUS enzyme activity in transgenic seedlings subjected to various hormonal treatments. The data are presented as the average of three independent biological replicates, with error bars indicating the standard deviation from the mean. Each replicate consisted of six in vitro cultured cassava seedlings. An asterisk (**) denotes statistically significant differences from the control group at a *p*-value of less than 0.01 (*t*-test).

To investigate the roles of the *MeAHL31* promoter in response to MeJA, ABA, and GA3 signals, transgenic *Arabidopsis* seedlings were exposed to 50–100 µM MeJA, ABA, and GA3, respectively. Subsequently, GUS activities were measured and contrasted. The MeJA treatment highly promoted the GUS activity (Figure 7A). Interestingly, a 50 µM MeJA treatment resulted in a more pronounced enhancement of GUS activity compared to the 100 µM MeJA treatment (Figure 7A). In contrast, treatments with ABA and GA3 did not elicit significant changes in GUS activity (Figure 7B). The results indicated that the *MeAHL31* promoter was positively modulated by MeJA, whereas ABA and GA3 exerted minimal influence on its activity.

## 3. Discussion

In recent years, cassava has emerged as a versatile crop with applications in various industries, including feeds, papermaking, pharmaceuticals, plastic processing, textiles, and biofuels [35]. However, the impact of global climate change has heightened the vulnerability of cassava to abiotic stresses, underscoring the need to identify genes and promoters that can enhance stress tolerance. Despite the growing importance of cassava, there is still a significant lack of genetic resources to improve its resilience.

Phylogenetic analysis has established the evolutionary connections between the MeAHL31 protein and those of other species. From Figure 1C, it can be observed that the MeAHL31 protein shares the closest homology with the AHL proteins from *Hevea brasiliensis*. This may be due to the fact that they both belong to the Euphorbiaceae family. However, research on HbAHL proteins is currently scarce. Based on the existing research, the *AHL* family has been extensively implicated in plant stress adaptation. In *Oryza sativa*, twenty *AHL* genes have been identified and shown to be up-regulated under salt and drought stress, indicating their role in stress signal transduction [19]. The identification of the *AHL* gene family in *Liriodendron chinense* suggested that *LcAHL* genes were involved in drought resistance [22]. *PtrAHL14* and *PtrAHL17* improved the cold tolerance of *Poncirus trifoliata* [17]. Genes exhibit distinct functions based on their tissue-specific expression patterns. In *Populus trichocarpa*, *PtrAHL34* showed significant expression in the root system. It was observed that under drought stress conditions, the expression of *PtrAHL34* was up-regulated at the 6-h interval, and then it gradually decreased, reaching a level akin to the normal level at 24 h [15]. Within the *BrAHL* gene family, *BrAHL16* was found to be highly expressed in the roots. Its expression was notably increased under osmotic stress conditions at 4 h, after which it was observed to decrease at subsequent time points [20]. Our study identified significant transcript level changes of *MeAHL31* in cassava in response to various stresses (Figure 4), suggesting its potential as a target gene for developing stress-resistant cassava.

Subcellular localization is a critical determinant of protein function. In plants, most AHL proteins were reported to be localized in the nucleus [36]. Our study revealed that the MeAHL31 protein is localized in both the cytoplasm and nucleus, suggesting it may participate in different biological processes in the nucleus and cytoplasm.

Promoter sequences are crucial for the precise regulation of gene transcription in plants. *Cis*-acting elements within promoters dictate the patterns of transcriptional activity in response to diverse cues [23]. Many studies have highlighted the presence of stress and hormone-related elements in *AHL* promoters [20,21]. In *Brassica rapa*, most of the *cis*-acting elements in the promoters of *BrAHL* genes are associated with abiotic stresses and hormonal responses [20]. Over 90% of these *BrAHL* genes possess plant hormone-responsive elements, such as those for MeJA, ABA, GA, and SA [20]. In tomato, the *cis*-acting elements of the promoter sequence among 18 *SlAHL* genes are mostly associated with hormone responsiveness, including ABA, GA, MeJA, auxin, and SA [21]. In this study, the *MeAHL31* promoter was found to contain ABA-responsive (ABRE), drought-responsive (MBS), MeJA-responsive (CGTCA and TGACG motifs), and GA-responsive (GARE-motif) elements (Table 1), indicating the plant’s ability to respond to these signals and adapt to environmental challenges. In transgenic *Arabidopsis*, the expression of the *MeAHL31* promoter was observed in most tissues and organs except seeds (Figure 5), suggesting its potential as a valuable tool in both basic research and applied biotechnology. We hypothesize that the *MeAHL31* promoter is highly stable, enabling it to maintain gene expression under various conditions. Consistent with the expression patterns, the promoter’s activity was enhanced by NaCl, drought, and MeJA stresses but not significantly under GA3 and ABA treatments (Figure 6 and Figure 7).

Plant hormones, particularly ABA, GA, and MeJA, are known to modulate plant responses to abiotic stresses [37]. Overexpression studies and hormone treatments have provided insights into the mechanisms by which these hormones enhance stress tolerance [38]. For example, GA3 has been shown to improve salt tolerance in plants [39], while MeJA activates antioxidant systems, thereby reducing oxidative stress [40]. These findings highlight the intricate regulatory network that hormones control in response to stress, suggesting potential strategies for bolstering crop resilience and boosting agricultural productivity under adverse environmental conditions. Ongoing research may uncover novel insights, advancing the harnessing of plant hormones for sustainable agriculture. In our study, the activity of the *MeAHL31* promoter was enhanced under NaCl, drought, and MeJA stresses, which hypothesize that *MeAHL31* may be regulated by the MeJA signaling pathway in response to abiotic stresses such as salt and drought. To substantiate this hypothesis, further research is required to clarify the underlying regulatory mechanisms. Our future work will focus on elucidating the regulatory role of the MeJA hormone in modulating the response of *MeAHL31* to abiotic stresses, aiming to enhance the understanding of stress tolerance pathways and contribute to sustainable agriculture practices.

## 4. Materials and Methods

### 4.1. Plant Materials and Growth Conditions

The South China 8 (SC8) variety of cassava was utilized in this research, which was cultivated in Lingao, Hainan Province, China. A range of tissues from the SC8 cassava plants, including terminal and axillary buds, petioles, young and mature leaves, stems, and both fibrous and tuber roots, were harvested after 180 days of growth. Post-harvest, the collected samples were promptly immersed in liquid nitrogen for rapid freezing and then stored at a temperature of −80 °C to preserve their integrity for subsequent RNA extraction.

### 4.2. Gene Structure and Phylogenetic Tree Analysis

The gene structure of *MeAHL31* was analyzed using GSDS version 2.0. The phylogenetic tree was constructed using MEGA 7.0 software based on the neighbor-joining (NJ) approach, followed by 1000 bootstrap replicates.

### 4.3. DNA Extraction and Promoter Cloning of MeAHL31

Genomic DNA was harvested from cassava seedlings utilizing the Plant Genomic DNA Kit (FOREGENE, Chengdu, China). The specific primer set designated as proMeAHL31-F/R was formulated for the amplification and cloning process of the MeAHL31 promoter sequence, utilizing the Primer Premier 6.0 software for sequence-specific design (details of which can be found in Supplementary Table S2). The *MeAHL31* promoter was isolated using Tks Gflex^TM^ DNA polymerase (TaKaRa). The PCR protocol is executed in accordance with the manufacturer’s guidelines for the specific enzyme used. Subsequently, the amplified DNA fragment was ligated into the pCAMBIA1304-35S-GUS::GFP vector (Figure S2).

### 4.4. Subcellular Localization of MeAHL31 Protein

The coding sequence (CDS) of MeAHL31, excluding the stop codon, was cloned via PCR using the 1300-MeAHL31-F/R primers, which were designed to incorporate recognition sites for the Spe I and BamH I enzymes (Table S2). The PCR-amplified fragment was subjected to double enzyme digestion and then ligated into the pCAMBIA1300-35S-GFP vector using T4 DNA ligase, resulting in the formation of the recombinant plasmid pCAMBIA1300-35S-MeAHL31::GFP. Using the Agrobacterium-mediated transient expression method, the fusion vector pCAMBIA1300-MeAHL31-GFP and the empty control vector pCAMBIA1300-35S-GFP were transformed into onion epidermal cells using Agrobacterium strain GV3101 in a competent state [41]. The GFP fluorescence (excitation 488 nm, emission 500–550 nm) and DAPI (excitation 360 nm, emission 460 nm) were observed under a confocal microscope (LEICA, TCPSP8, Wetzlar, Germany).

### 4.5. Stress and Hormonal Treatments for Cassava

SC8 seedlings were grown in a temperature-controlled chamber (Haikou, China), and kept at 28 °C, 16 h photoperiod, 60% relative humidity. The 30-day-old seedlings were removed from the Murashige and Skoog (MS) medium and sprayed and immersed into 100 mM mannitol, then 300 mM NaCl solutions for 0 h, 2 h, 6 h, 12 h, 24 h, and 48 h. In the same way, the 30-day-old seedlings were removed from the MS medium, sprayed, and immersed into 100 µM MeJA, 100 µM ABA, and 100 µM GA3 solutions for 0 h, 2 h, 6 h, 12 h, and 24 h. Following the treatments, the roots, stems, and leaves of the seedlings were sampled and frozen in liquid nitrogen, and then stored at −80 °C for subsequent DNA and RNA extractions.

### 4.6. RNA Extraction and qRT-PCR Analysis for MeAHL31

Total RNA was extracted from the cassava seedlings using the RNA Total Isolation Kit Plus (FOREGENE, Chengdu, China). Then, 1 µg of RNA was employed for cDNA synthesis with the RTIII All-in-One Mix with dsDNase (Monad, Suzhou, China). The specific primer pairs (qMeAHL31-F/R) for qRT-PCR were designed using Primer Premier 6.0 software (Table S2). The qRT-PCR amplification reactions were performed on a Roche LightCycle 96 Instrument, using the MonAmp ChemoHS qPCR Mix (Monad, Suzhou, China). The thermal cycling protocol was as follows: 95 °C for 30 s, followed by 40 cycles of 95 °C for 10 s, 60 °C for 10 s, and 72 °C for 30 s. Each experiment was conducted with three technical replicates. The relative quantification of *MeAHL31* transcripts was determined using the 2^−ΔΔCT^ method [42]. A *t*-test was applied to determine significant differences at a confidence level of *p* < 0.01.

### 4.7. Cis-Acting Element Analysis of MeAHL31 Promoter

The sequence, number, location, and function of abiotic stresses and hormone-responsive elements within the *MeAHL31* promoter were analyzed using the PlantCARE database [43].

### 4.8. Vector Construction of MeAHL31 Promoter and Generation of Transgenic Arabidopsis 

The pCAMBIA1304-*proMeAHL31*-GUS::GFP vector was transformed into *Arabidopsis* by the *Agrobacterium*-mediated floral dipping method [44]. The transformed lines were selected on 1/2 MS medium supplemented with 50 mg/L hygromycin (Hyg). The homozygous T3 generation of transgenic *Arabidopsis* seeds was used for further experiments. The total DNA of the T3 generation of transgenic *Arabidopsis* seeds was used as a template and 1304-F/R as primers to identify transgenic lines (Table S2). 

### 4.9. Stress and Hormone Treatments for Transgenic Arabidopsis 

The pCAMBIA1304-proMeAHL31-GUS::GFP transgenic seeds were disinfected with 75% ethanol and air-dried on filter paper before being sown on 1/2 MS medium. After stratification at 4 °C in the dark for three days to facilitate germination, the seeds were then transferred to a growth chamber (Haikou, China) with conditions set at 22 °C, a 16-h photoperiod, and 60% relative humidity for further cultivation for 5 days. Subsequently, the 5-day-old seedlings were transferred to 1/2 MS medium plates that included a range of concentrations for stress and hormone treatments: 100–200 mM NaCl, 50–100 mM mannitol, 50–100 µM MeJA, 50–100 µM ABA, and 50–100 µM GA3. Each treatment was applied for 7 days. Seedlings grown on regular 1/2 MS medium served as the control. Each treatment was replicated six times. Following the stress and hormone treatments, the transgenic *Arabidopsis* seedlings were subjected to GUS staining and enzymatic activity assays.

### 4.10. GUS Staining and Activity Detection in Transgenic Arabidopsis

GUS staining and activity detection in the transgenic *Arabidopsis* seedlings were conducted as described previously [45,46,47]. After staining, the seedlings were photographed using the Keyence Ultra Depth of Field Microscope. GUS activities were analyzed and compared based on a *t*-test, at a significant level of *p* < 0.01.

### 4.11. Statistical Analysis

The data are presented as the mean ± SD, and the data of three independent experiments were analyzed with a one-way analysis of variance. The value *p* ≤ 0.01 was considered significant with GraphPad Prism 8 software.

## Figures and Tables

**Table 1:** Sequence, number, location, and function of *cis*-acting elements in the *MeAHL31* promoter.

Element Name	Core Sequence	Number	Location (bp)		Function
			(+) Sense Strand	(−) Antisense Strand	
ABRE-element	ACGTG	1		−707	ABA-responsive
CGTCA-motif	CGTCA	1		−1219	MeJA-responsive
TGACG-motif	TGACG	1	+1219		MeJA-responsive
MBS	CAACTG	1		−973	Drought-responsive
GARE-motif	TCTGTTG	1		−583	GA-responsive

## Data Availability

Data is contained within the article and Supplementary Materials.

## Supplementary Materials

The following supporting information can be downloaded at: https://www.mdpi.com/article/10.3390/ijms25147714/s1.
