# Supplementary material for: Promoter of Cassava MeAHL31 Responds to Diverse Abiotic Stresses and Hormone Signals in Transgenic Arabidopsis
Source: Int J Mol Sci. 2024 Jul 14;25(14):7714. doi: 10.3390/ijms25147714 (PMC11276720; doi:10.3390/ijms25147714)
Supplement: Supplementary file 1 [file ijms-25-07714-s001.zip › Supplementary Figures.pdf]

Supplementary Date

Supplementary Figures

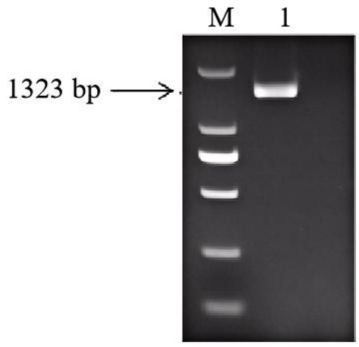

Fig.S1 Clone of *MeAHL31* promoter. M: 2000 DNA marker; 1: *proMeAHL31*

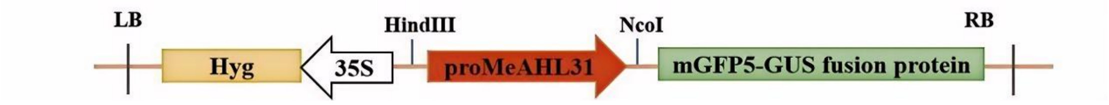

Fig.S2 Schematic diagram of pCAMBIA1304-*proMeAHL31*-GUS::GFP vector construction; LB indicates the left border and RB indicates the right border of the pCAMBIA1304-*proMeAHL31*-GUS::GFP vector

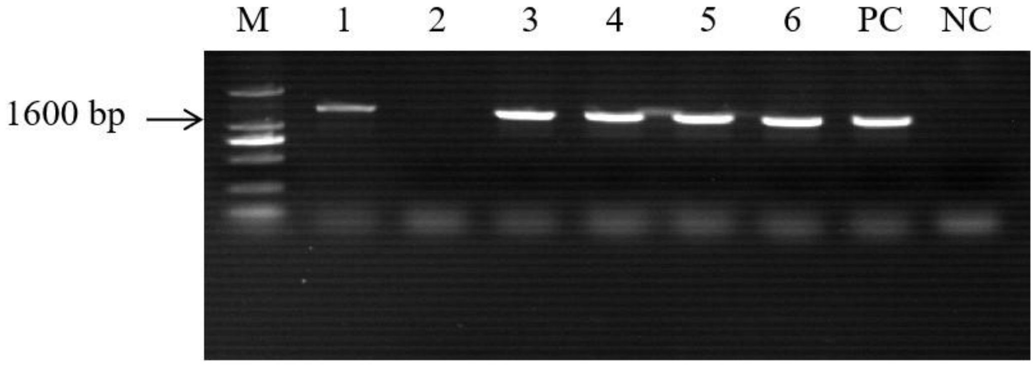

Fig.S3 PCR identification of the *proMeAHL31* transgenic plants. M: 2000 DNA marker; 1-6: transgenic plants; PC: positive control; NC: negative control
